# Analysis of microRNAs in Small Urinary Extracellular Vesicles and Their Potential Roles in Pathogenesis of Renal ANCA-Associated Vasculitis

**DOI:** 10.3390/ijms23084344

**Published:** 2022-04-14

**Authors:** Jana Frydlova, Iveta Zednikova, Veronika Satrapova, Eva Pazourkova, Sarka Santorova, Zdenka Hruskova, Vladimir Tesar, Martin Vokurka, Petr Prikryl, Marie Korabecna

**Affiliations:** 1 Institute of Pathological Physiology, First Faculty of Medicine, Charles University, U Nemocnice 5, 128 53 Prague, Czech Republic; jana.frydlova@lf1.cuni.cz (J.F.); martin.vokurka@lf1.cuni.cz (M.V.); 2 Institute of Biology and Medical Genetics, First Faculty of Medicine, Charles University and General University Hospital, Albertov 4, 128 00 Prague, Czech Republic; iveta.zednikova@lf1.cuni.cz (I.Z.); eva.pazourkova@lf1.cuni.cz (E.P.); sarka.santorova@vfn.cz (S.S.); 3 Department of Nephrology, First Faculty of Medicine, Charles University and General University Hospital, U Nemocnice 2, 128 08 Prague, Czech Republic; veronika.satrapova@vfn.cz (V.S.); zdenka.hruskova@vfn.cz (Z.H.); vladimir.tesar@vfn.cz (V.T.)

**Keywords:** ANCA-associated vasculitis (AAV), microRNA, next-generation sequencing, quantitative PCR, proteomics, biological pathways, pathogenesis

## Abstract

Antineutrophil cytoplasmic antibodies (ANCA)-associated vasculitis (AAV) represents an autoimmunity disease characterized by high mortality. For successful treatment, the detailed knowledge of its complex pathogenesis and the set of biomarkers for differential diagnostics are desired. Analysis of molecular content of small urinary extracellular vesicles (uEV) offers the possibility to find markers in the form of microRNAs (miRNAs) and study the pathways involved in pathogenesis. We used next-generation sequencing in the first preliminary study to detect the miRNAs with altered expression in uEVs of patients with AAV in comparison with age-matched controls. We confirmed the results using single-target quantitative polymerase chain reaction tests on different sets of samples and found five miRNAs (miR-30a-5p, miR-31-3p, miR-99a-5p, miR-106b-5p, miR-182-5p) with highly elevated levels in uEVs of patients. We performed the comparison of their targets with the differentially expressed proteins in uEVs of patients included in the first phase. We realized that upregulated miRNAs and proteins in uEVs in AAV patients target different biological pathways. The only overlap was detected in pathways regulating the actin cytoskeleton assembly and thus potentially affecting the glomerular functions. The associations of upregulated miRNAs with pathways that were neglected as components of complex AAV pathogenesis, e.g., the epidermal growth factor receptor signaling pathway, were found.

## 1. Introduction

Antineutrophil cytoplasmic antibodies (ANCA)-associated vasculitis (AAV) is characterized by necrotizing inflammation of small vessels of the kidney, lungs or ear, nose, and throat organs. Myeloperoxidase (MPO) and proteinase-3 (PR3) serve as the two major antigens. MPO and PR3 are stored in cytoplasmic granules of neutrophils, where they are sheltered from the immune system [1]. They can be released by the process of NETosis, which may be stimulated by a broad spectrum of pathophysiological factors associated with different diseases [2]. During NETosis, the neutrophil extracellular traps containing nuclear DNA release MPO and PR3 into extracellular space. After neutrophil degranulation, the PR3 is inactivated by α1-antitrypsin and MPO by ceruloplasmin, and thus the development of autoantibodies is prevented. Ineffective removal of MPO and PR3 may induce the development of ANCA [1], which activates monocytes and neutrophils. Activated neutrophils degranulate, undergo the process of NETosis, increase the expression of adhesion molecules, and generally provide the spectrum of proinflammatory stimuli [3]. All these processes result in serious damage of endothelium integrity, in which endothelial cells detach and ectodomains of membrane-bound proteins are cleaved [3]. Pathophysiological mechanisms involved in complex AAV pathogenesis are comparatively unknown. Molecular analysis of the content of extracellular vesicles (EVs) found in urine can help to get new insights into this very complex issue. EVs including exosomes are released into urine from each segment of the nephron [4]. Exosomes carry biologically active molecules, including microRNAs (miRNAs) and proteins, and therefore they can serve as messengers in intercellular communication and regulate tissue microenvironment affecting the signaling pathways in target cells [5]. In addition to these functions, the EVs may play a role in the removal of excessive molecules from cells of their origin and thus provide the information about pathological conditions in tissues [6,7].

Exosomes belong to extracellular nanovesicles having a diameter of 30–150 nm [8]. The term “small extracellular vesicles” is now widely accepted by the international community regarding the challenging methodologies associated with their isolation from biological fluids [9].

The role of EVs and their cargo in the pathogenesis of vasculitis and glomerulonephritis has been rarely studied with the exception of the focus on EVs derived from blood [10]. Recently, it was demonstrated that the miRNAs contained in urinary EVs (uEVs) may serve as useful biomarkers of disease activity and fibrosis as reported in lupus nephritis [11,12].

The miRNAs in uEVs had not yet been examined in association with their role in the pathogenesis of AAV, and therefore we conducted this study. In the first phase, we employed next-generation sequencing (NGS) to obtain the profiles of all miRNA types detectable in the uEVs of AAV patients and age-matched healthy controls. In the confirmation phase, we performed single-target quantitative real-time polymerase chain reaction (RT-PCR) tests for 28 miRNAs found as the most elevated in patients using sequencing technology. We used a very stringent approach for normalization; hence, we validated only the results for five miRNAs (miR-30a-5p, miR-31-3p, miR-99a-5p, miR-106b-5p, and miR-182-5p) having the most elevated levels in uEV. Using bioinformatics analysis, we compared the targets of these miRNAs with the sets of proteins with altered abundancy in uEVs that were found through proteomic analysis performed on the identical set of samples in our NGS-based phase and published earlier [13]. We realized that highly abundant miRNAs and proteins, both included in uEVs in AAV patients, regulate different biological pathways. The bioinformatic analysis highlighted the association of upregulated miRNAs with biological pathways that were neglected as components of complex AAV pathogenesis despite the existence of clinical evidence, e.g., an epidermal growth factor receptor (EGFR) signaling pathway [14]. We found that the five miRNAs highly elevated in uEVs can potentially regulate the processes on cell surfaces and signalization events and act in accord with some differentially expressed proteins in uEVs [13]. The miRNAs identified by us for potential differential diagnostics of AAV should be explored further to clarify their roles in AAV pathogenesis and their association with disease activity and progression.

## 2. Results

### 2.1. Next Generation Sequencing Based Phase

**Figure 1:**
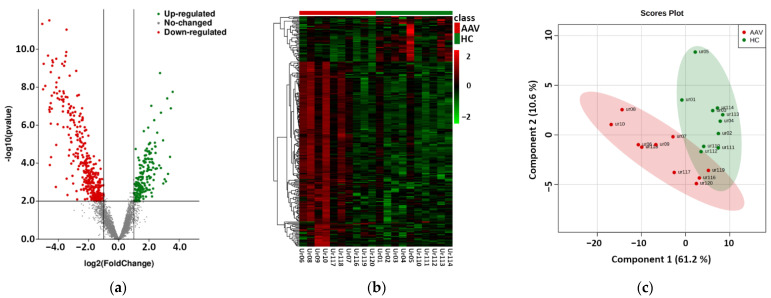
Results of the phase based on next-generation sequencing of urinary extracellular vesicle-derived miRNAs of 10 antineutrophil cytoplasmic antibodies-associated vasculitis (AAV) patients vs. 10 healthy controls (HC). (**a**) Volcano plot of the differential expression analysis of mapped intraluminal miRNAs, (**b**) Hierarchical clustering heatmap of differentially expressed miRNAs in uEVs (detailed data and high-resolution map shown in Supplementary Table S1 and Figure S1), (**c**) Partial least-squares—discriminant analysis of differentially expressed miRNAs in uEVs.

Raw reads were clipped to adapter sequences and mapped to a database of human miRNA. A total of 2538 miRNAs were detected in analyzed samples. Unmapped reads were used for prediction of new miRNAs. This way, 1900 potential new miRNAs were identified. All reads were mapped to a set of reference sequences combining known miRNAs and newly predicted miRNAs. The sum of mapped reads was processed using two specific packages in R as described in the Methods section. In total, 161 differentially expressed miRNAs were revealed using DESeq2, and 238 differentially expressed miRNAs were revealed using edgeR (Table S1). All 161 miRNAs detected by DESeq2 were also found by edgeR. The results of this phase are summarized in Figure 1. We observed significant differences in the expression of uEV-derived miRNAs between control and patient groups, as shown by the volcano plot of differential expression analysis, the hierarchical clustering heatmap, and the partial least-squares discriminant analysis, which explained more than 70% of the total variance.

All differentially expressed miRNAs found in this phase together with those selected for RT-PCR confirmation phase are listed in Table S1. We selected 19 miRNAs with elevated levels in uEVs in patients for the confirmation phase. The nine miRNAs potentially suitable for a normalization procedure were selected using the next-generation sequencing data and geNorm algorithm [15]: miR-182-5p, miR-4503, miR-711, miR-498, miR-6724-5p, miR-558, miR-4326, miR-1305, and miR-574-3p (Table S1) and included in the confirmation phase experiments.

### 2.2. RT-PCR Based Confirmation Phase

The selection of proper miRNAs or a group of them to normalize experiment results represents the most challenging but crucial step in studies dealing with the evaluation of differential expression of these molecules. There are some well-established procedures, but the choice of one of them must be carefully evaluated for each study separately [16].

**Figure 2:**
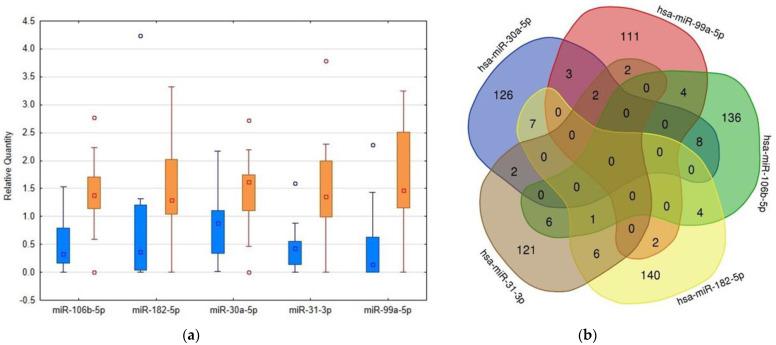
Comparison of levels of RT-PCR confirmed microRNAs in urinary extracellular vesicles (uEVs) in patients and controls and detection of their overlapping targets. (**a**) Box plots created using Statistica software version 12 (StatSoft, Inc., Tulsa, OK, USA) showing the differences in microRNA abundance in uEVs between patients and controls, quantities related to global mean, medians given by squares, 25th and 75th percentiles given by rectangles, outliers given by circles, blue columns—control subjects, orange columns—patients. (**b**) Venn diagram demonstrating the overlaps among the targets of selected microRNAs. The targets were detected using the mirDIP4.1 database with the highest score of reliability.

The nine miRNAs mentioned above were selected as potentially suitable for normalization during the NGS-based phase, but the results of confirmatory experiments indicated they were unsuitable for this purpose. They were not detectable in all samples, and miR-182-5p was elevated in samples from patients according to the raw data evaluation. Therefore, we decided to normalize the results to the global mean of all miRNAs studied in this phase and accept the limitation associated with this approach, namely that only the miRNA most strikingly elevated would be detected as having an increased abundance in patients’ samples. This limitation can explain the findings of decreased abundance of miR-26a-5p, miR-192-5p, and miR-191-5p in uEVs in patients in contrast to next-generation sequencing data. Using this approach, we successfully confirmed the results of the NGS-based phase for 4 out of 19 miRNAs having elevated levels in uEVs in patients in this first phase, and we found a significantly increased amount of miR-182-5p, which was originally regarded as an miRNA suitable for result normalization. Nonsignificant differences between patients and controls were found in the remaining analyzed miRNAs: let-7f-5p, miR-10a-5p, miR-23b-3p, miR-27a-3p, miR-138-5p, miR-141-3p, miR-424-5p, miR-498, miR-558, miR-574-3p, miR-711, miR-1305, miR-3614-3p, miR-4326, miR-4503, miR-6724-5p, miR-6748-3p, and miR-8060. The results of the RT-PCR-based confirmation phase are represented in Figure 2 and Table 1. We used the standard deviations obtained from quantitative RT-PCR analysis to calculate post hoc power of this RT-PCR-based analysis using G Power software [17] (Table 1).

### 2.3. Comparison with Proteomic Results and Bioinformatic Analysis

**Figure 3:**
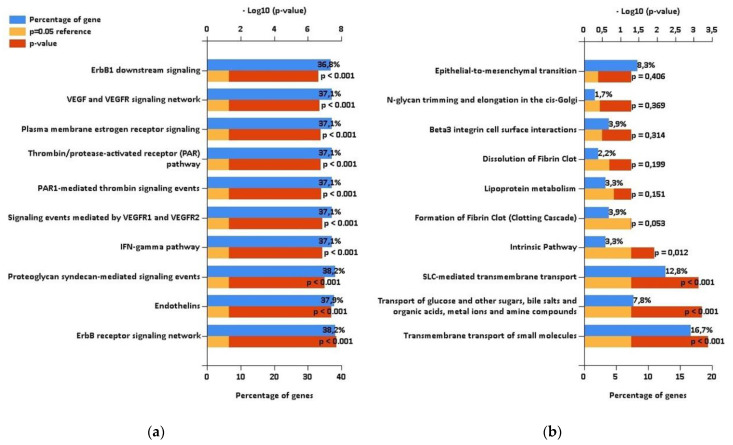
The results of enrichment analysis performed using FunRich. (**a**) Biological pathways regulated by targets of RT-PCR confirmed microRNAs with increased levels in urinary extracellular vesicles (uEVs) in antineutrophil cytoplasmic antibodies-associated vasculitis (AAV) patients. (**b**) Biological pathways regulated by proteins with decreased levels in uEVs in AAV patients according to proteomic analysis [13] performed on patients involved in the NGS-based phase of this study.

Using the mirDIP database, we detected that the 5 highly elevated miRNAs inside uEVs of AAV patients can regulate altogether the expression of 681 different proteins (Figure 2). Enrichment analysis of these targets showed significantly higher proportions of proteins involved in specific biological pathways, as demonstrated in Figure 3. This analysis especially highlighted the ErbB receptor signaling pathway and the proteoglycan syndecan mediated pathways as the targets of the analyzed miRNA set (Figure 3). In contrast, the identical analysis performed on sets of either down- or upregulated proteins detected in uEVs of patients involved in the NGS-based phase [13] revealed quite different sets of biological pathways.

**Figure 4:**
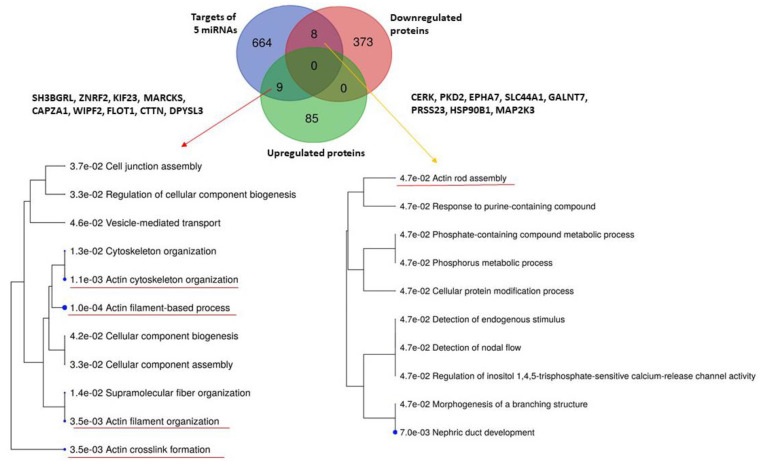
Analysis of biological processes regulated by five RT-PCR confirmed miRNAs and either up- or downregulated proteins in urinary extracellular vesicles detected in the study by Prikryl et al. [13]. Analysis was performed using database ShinyGOv.066.

While the downregulated proteins contained in uEVs of patients regulate significantly the transport of small molecules across the cell membrane (Figure 3), no significantly enriched biological pathways were found for upregulated proteins (Figure S2). We explored the biological processes regulated by proteins with decreased levels in uEVs and simultaneously with increased levels of miRNAs involved in the regulation of their expression. We found eight proteins acting namely in nephron development but also having the potential to affect the actin cytoskeleton assembly (Figure 4). Actin cytoskeleton organization is also significantly influenced by the activity of proteins with increased levels in uEVs, which simultaneously contain the miRNAs having the potential to downregulate the expression of these proteins in target cells (Figure 4).

## 3. Discussion

Generally, it is difficult to interpret the biological meaning of the elevated miRNA levels in uEVs. The miRNA profile detected in uEVs may mirror the conditions in cells of the tissue of their origin. Some of the miRNAs included in uEVs can represent the waste material only, but others can be involved in intercellular signalization.

It was experimentally proven that uEVs can mediate the intranephron communication. Tubular epithelial cells produce EVs that are involved in normal kidney physiology. Communication between the proximal and distal tubule is mediated by these EVs [18]. They can also ensure the communication with interstitial cells (fibroblasts and macrophages) and thus modify the immune response [19]. Glomerular mesangial cells and podocytes also release EVs. EVs from podocytes communicate with proximal tubule epithelial cells and can promote fibrotic signaling [20]. In acute vasculitis, the elevated levels of EVs derived from endothelial cells were observed [21].

The above-mentioned data were obtained in vitro. It is necessary to increase our knowledge about the conditions under which the different cell types produce uEVs and select their cargo [22].

The dominant proteins present in uEVs of healthy persons were characterized [23]. The most abundant miRNAs in uEVs in healthy individuals were also detected, and the uptake of uEVs by cultured renal epithelial cells was proven together with the lowered expression of target proteins of these miRNAs [24]. The miR-99a-5p, miR- 30a-5p, and miR 182-5p belong to the 50 most abundant miRNAs in uEVs detected by Gracia et al. [24], but none of the miRNAs described by us as elevated in uEVs of AAV patients belong to the set of miRNAs differentially expressed in uEVs of males and females [25].

First, we will discuss what is recently known about each of the miRNAs detected by us as having higher abundance in uEVs of AAV patients.

The role of miR-31-3p was evaluated in multiple studies dealing with the monitoring of anti-EGFR therapy in patients with colorectal cancer. The response rate was higher in patients with its lower expression [26]. The development of cutaneous vasculitis and formation of ANCA was described in breast cancer patients treated with EGFR inhibitors [14]. Our bioinformatic analysis highlighted the regulation of the EGFR signaling pathway as the part of multifaceted AAV pathogenesis (Figure 3) in accordance with these pieces of evidence.

The authors of [24] found miR-106b-5p among the 257 most abundant miRNAs in uEVs of healthy individuals, but its altered expressions were not reported in the field of nephrology [27,28,29,30,31,32,33,34,35,36,37].

Under physiological conditions, miR-99a-5p is abundant in uEVs [24]. Its increased levels were not reported in studies dealing with kidney diseases [27,28,29,30,31,32,33,34,35,36,37]. Cultured human podocytes after transfection with this miRNA downregulated the mammalian target of rapamycin (mTOR) protein expression and also downregulated the protein vimentin, which serves as a marker of podocyte injury [27].

According to [24], miR-30a-5p is the third-most abundant miRNA in uEVs of healthy individuals. Its elevated levels were reported in uEVs in nephrotic syndrome (NS) [28,29,30]. It may serve as a biomarker for evaluation of focal segmental glomerulosclerosis (FSGS) activity and treatment response [31]. In urinary sediment, the significantly elevated levels of this miRNA were found in IgA nephropathy, and their possible erythrocyte-derived origin was discussed [32]. The levels of this miRNA are downregulated in urinary exosomes in autosomal dominant polycystic kidney disease (ADPKD) [33]. In uEVs of patients with diabetic nephropathy (DN), significantly higher amounts of miR-30a-5p were detected [34]. Elevated levels of miR-30a-5p were also reported in the urine of patients with acute kidney injury caused by intoxication [35].

miR-182-5p was detected in elevated levels in tubular cells in association with ferroptosis—an iron-dependent form of cell death and association with acute kidney injury was reported [36]. This miRNA was identified as the main driver of post-transplantation acute kidney injury [37]. It was demonstrated that its inhibition ameliorates ischemic acute kidney injury [38] and contributes to attenuation of lupus nephritis [39]. Using the mouse model of ADPKD, it was reported that miR-182-5p regulates actin cytoskeleton rearrangement and promotes cystogenesis [40]. In [41], miR-182 was also found as the constant component of urine supernatants independently on the health status and sex of the urine donor.

We compared the panel of miRNAs detected by us as the most abundant in uEVs in AAV patients with the sets of miRNAs reported as differentially expressed in uEVs in other kidney diseases [28,29,30,31,33,34], and we found only one overlap, miR-30a-5p, which represents the highly abundant miRNA in uEVs [24] and was detected as elevated there in patients with NS [28,29,30], FSGS [31], and DN [34]. The elevated levels of other miRNAs included in our panel are unique for uEVs in AAV patients, and therefore their role in differential diagnostics deserves further study. In this context, it is important to mention that the methodology of exosome isolation and normalization of miRNA quantification plays a crucial role in the interpretation of results [16,42].

As miRNAs also can fulfill noncanonical roles associated with epigenetic regulation inside the nucleus and with regulation of transcription [43], it is practically impossible to completely describe the role of a set of miRNAs.

In addition to the role of EGFR signaling in the AAV pathogenesis, our bioinformatic analysis also highlighted the inclusion of vascular endothelial growth factor (VEGF) signalization among the biological pathways regulated by the targets of miRNAs with elevated levels in uEVs in patients (Figure 3). This association was mentioned only in one case report, where the correlation between serum VEGF levels with C-reactive protein and anti-MPO titer was clearly demonstrated [44].

Venous thromboembolism was observed with high prevalence in AAV patients, and its association with disease activity was proposed [45]. It is in accordance with our finding of the “Thrombin/protease activated pathway” among pathways significantly regulated by targets of miRNAs selected by us. Inclusion of “Proteoglycan syndecan-mediated signaling events” among these pathways corresponds with the fact that syndecan represents a major protein expressed on the glycocalyx of endothelial cells and its serum levels mirror vascular endothelial damage and inflammation in acute systemic vasculitis—Kawasaki disease [46].

“The interferon–gamma pathway” also belongs to pathways detected as significantly affected by the targets of our miRNA set. The number of double negative (CD4^−^CD8^−^) T-lymphocytes was increased in patients with AAV, and an increased percentage of these cells expressing interferon was found [47].

Endothelins are known as important regulators of vascular functions in renal physiology [48]; therefore, their inclusion among the proteins regulated by our miRNA set is also in agreement with supposed mechanisms of AAV pathogenesis.

When comparing the targets of selected miRNAs and proteins found to be up- or downregulated in uEVs in AAV [13], we found some overlaps (Figure 4). The analysis of biological processes affected by these proteins revealed mainly the functions of actin cytoskeleton. It was reported that the expression of alfa-small muscle actin in glomeruli is associated with AAV progression [49]. The presence of ANCA may also contribute to the F-actin polymerization in neutrophils and allow them to reach the small capillaries where they can undergo the process of NETosis [50].

In conclusion, we detected five miRNAs with significantly elevated levels in uEVs, and the bioinformatic analysis of their targets in association with the result of a proteomic study [13] led to the meaningful interpretation of potential roles of these miRNAs in very complex AAV pathogenesis. Further comparative studies that would also include patients with other proliferative glomerular diseases and would focus on the clarification of potential pathogenic roles of these miRNAs are needed regarding the practical application of this miRNA set in differential diagnostics and disease-progression monitoring.

## 4. Materials and Methods

### 4.1. Patients and Urine Sample Collection

The ethical committee of the General University Hospital in Prague approved the study in accordance with the Declaration of Helsinki (93/14 Grant VES 2015 AZV 1. LF UK). All patients were recruited from the Department of Nephrology of First Faculty of Medicine at Charles University and General University Hospital in Prague, Czech Republic, and signed informed consent. In the initial phase, ten individual biological replicates of patients with renal biopsy-proven AAV versus ten healthy control subjects were analyzed using the NGS. The obtained results were then confirmed by the quantitative RT-PCR method. In this confirmation phase, 14 extra consecutive patients with AAV, willing to participate, and 6 extra healthy controls were added to the original groups, so the confirmation groups were then represented by 24 patients with renal biopsy-proven AAV and 16 healthy donors (Table 2).

The first-morning urine of the midstream void was collected in a sterile container on the day of renal biopsy, mixed, and immediately centrifuged at 3000× *g* for 30 min at 20 °C to remove cells, bacteria, cellular casts, and the bulk of the polymeric chains of uromodulin. The obtained supernatants were supplemented with protease inhibitors (Roche, Mannheim, Germany), aliquoted, and stored at −80 °C.

### 4.2. Urine Sample Preparation

The pH of thawed urine samples was adjusted using HEPES buffer pH 8.5 at a final concentration of 50 mM and 10 mM EDTA. Samples were then centrifuged at 10,000× *g* for 30 min at 20 °C. The supernatant was treated by proteinase K (New England Biolabs, Ipswich, UK) at enzyme-to-protein ratio 1:50 for 1 h at 37 °C in presence of 20 mM dithiothreitol, filtered through 0.22 μm Steriflip (EMD Millipore, Burlington, MA, USA). The flowthrough was collected and supplemented with protease inhibitors.

### 4.3. Urinary Extracellular Vesicle Enrichment and miRNA Isolation

The 30 mL of pre-processed urine was transferred to a sterile conical polypropylene tube (Beckman Coulter, Brea, CA, USA) and ultracentrifuged at 100,000× *g* (avg) (28,000 RPM, k-Factor = 246) in a SW-28 rotor for 120 min at 20 °C to obtain a crude EV pellet. The pellet was resuspended in 100 μL of 10 mM Tris-HCl pH 7.6 with 2.5 mM MgCl_2_ and 0.5 mM CaCl_2_ including 2 units of DNAse I (New England Biolabs, Ipswich, UK) and incubated for 20 min at 37 °C. The resulted solution was then diluted with PBS and recentrifuged again at 150,000× *g* for 120 min at 20 °C. This washing step was repeated, and the final uEV pellet was collected and resuspended in 50 μL of PBS for subsequent miRNA extraction isolation. Exosomal miRNAs were isolated using a Urine microRNA Purification Kit (Norgen Biotek, Thorold, ON, Canada) according to manufacturer protocol, and purified miRNA samples were placed at –80 °C.

### 4.4. Next Generation Sequencing Based Phase

Preparation of miRNA libraries, quality control, and sequencing were carried out according to the appropriate manufacturer’s kit protocols listed below. MiRNA libraries were prepared using an Ion Total RNA-Seq Kit v2 (Thermo Fisher Scientific, Waltham, MA, USA). The libraries were then quality-controlled using a 2100 Bioanalyzer Instrument with High Sensitivity DNA Kit (Agilent, Santa Clara, CA, USA) and quantified with a KAPA library Quantification Kit (KAPA Biosystems, Wilmington, MA, USA). Template preparation was done using an Ion PI Hi-Q OT2 200 Kit (Thermo Fisher Scientific). The sequencing was carried out using an Ion Proton system with an Ion PI Hi-Q Sequencing 200 (Thermo Fisher Scientific) with the following settings: single end, 200 bp, 60–80 million reads per chip. The successful sequencing run provided output of around 30 million high-confidence single-end reads with average length from 26 to 37 bp.

The quality of raw reads was evaluated using FastQC (v0.11.5) and MultiQC (1.0.dev0). Removing, trimming or correcting reads that did not meet the defined standards was performed using Trimmomatic Version 0.36. Clipping adaptor sequences were carried out using cutadapt (v1.9.1). The trimmed reads were aligned to the human miRNA reference using BLAST engine for local alignment (v2) with word size setting 12. Unmapped reads were de novo assembled using Velvet (v1.2.09) assembler with kmer set to 29. The potential new miRNAs were predicted from assembled contigs. Unmapped reads were aligned to predicted new miRNAs. The number of mapped reads to human miRNA references and potential new miRNA references were extracted and used for differential gene expression analysis, which was performed using DESeq2 and EdgeR. The miRNA with logfold change more than 2 or less than −2 and with *p*-value less than 0.05 were considered significantly differentially expressed.

### 4.5. Single Target RT-PCR Based Confirmation Phase

Reverse transcription of purified miRNAs was performed in accordance with the protocol of a universal TaqMan™ Advanced miRNA cDNA Synthesis Kit (Applied Biosystems). Expressions of individual miRNAs were determined via a quantitative RT-PCR method using TaqMan™ Advanced miRNA Assays and TaqMan™ Fast Advanced Master Mix (Applied Biosystems). The plates with assays were prepared using the automated liquid handling system epMotion 5075 (Eppendorf). All reactions were run in triplicates on the QuantStudio 12K Flex Real-time PCR System. Results were processed in ExpressionSuite Software v1.0.3 (Applied Biosystems™, Carlsbad, CA, USA), qBase+© v2.4 (Biogazelle, Gent, Belgium) and Statistica software version 12 (StatSoft, Inc., Tulsa, OK, USA). Comparison of the miRNAs’ expressions between patients and controls was performed with Mann–Whitney’s U-test with the Benjamini–Hochberg correction to multiple testing (*p*-value ≤ 0.05; fold change ≥ 2).

### 4.6. Bioinformatic Analysis

The targets of miRNAs were detected using mirDIP 4.1 [51]. Enrichment analysis to find the significantly affected biological pathways and processes was performed by FunRich [52] and ShinyGO [53]. Hierarchical clustering and partial least-squares discriminant analysis were carried out using the MetaboAnalyst v5.0 software tool [54]. Venn diagrams were constructed using a freely available online tool [55].

## Figures and Tables

**Table 1:** Results of the RT-PCR-based confirmation phase. The global mean method was used for normalization, *p*-values were calculated using Mann–Whitney’s U-test with the Benjamini–Hochberg correction, FC (P/C)—Fold change, patients compared to control subjects, only FC > 2 was regarded as relevant, SD—standard deviation, Power—calculated post hoc for RT-PCR-based confirmation phase of this pilot study.

miRNA	*p*-Value	FC (P/C)	SD (Controls)	SD (Patients)	Power
miR-26a-5p	5.982 × 10^−6^	0.422	0.276	0.053	1.0000
miR-192-5p	5.417 × 10^−4^	0.312	0.546	0.099	1.0000
miR-191-5p	3.871 × 10^−2^	0.720	0.182	0.077	1.0000
**miR-31-3p**	**2.439 × 10^−4^**	**2.933**	0.166	0.232	1.0000
**miR-106b-5p**	**4.253 × 10^−4^**	**2.841**	0.124	0.214	1.0000
**miR-99a-5p**	**4.257 × 10^−3^**	**4.329**	0.161	0.282	1.0000
**miR-30a-5p**	**9.190 × 10^−3^**	**2.551**	0.154	0.172	1.0000
**miR-182-5p**	**1.348** **× 10^−2^**	**3.175**	0.190	0.171	1.0000
miR-24-3p	2.075 × 10^−2^	1.412	0.071	0.073	1.0000
miR-200a-3p	3.871 × 10^−2^	1.422	0.095	0.171	0.9999

**Table 2:** Characteristics of patients and control subjects.

Parameters	NGS Initial Phase		RT-PCR Confirmation Phase	
	AVV Patients *n* = 10	Healthy Controls *n* = 10	AVV Patients *n* = 24	Healthy Controls *n* = 16
Sex (Male/Female)	6/4	6/4	12/12	8/8
Age (median, range, years)	65.5 (37–74)	55 (42–74)	63.5 (21–78)	57 (42–74)
S-creatinine (median, range, μmol/L)	241.5 (113.1–438.3)	79.1 (64.4–86.1)	302.0 (52.3–480.1)	82.2 (64.4–91.3)
Proteinuria (median, range, g/24 h)	1.62 (0.21–3.60)	0.05 (0.03–0.13)	1.44 (0.11–3.60)	0.05 (0.03–0.13)
Hemoglobin (median, range, g/L)	91.0 (79–142)	n/a	98.5 (77–142)	n/a
C-reactive protein (median, range, mg/L)	103.5 (1.1–153.0)	n/a	12.0 (1.1–153.0)	n/a
Organ involvement (kidney/lung/ENT/eye/skin)	10/3/0/1/0	n/a	24/9/4/1/2	n/a
ANCA-subtypes (PR3/MPO/neg.)	3/7	n/a	7/15/2	n/a

## Data Availability

The data presented in this study are available on request from the corresponding author. The data are not publicly available.

## Supplementary Materials

The following are available online at https://www.mdpi.com/article/10.3390/ijms23084344/s1.
