# Supplementary figures and images for: Analysis of microRNAs in Small Urinary Extracellular Vesicles and Their Potential Roles in Pathogenesis of Renal ANCA-Associated Vasculitis
Source: Int J Mol Sci. 2022 Apr 14;23(8):4344. doi: 10.3390/ijms23084344 (PMC9028884; doi:10.3390/ijms23084344)

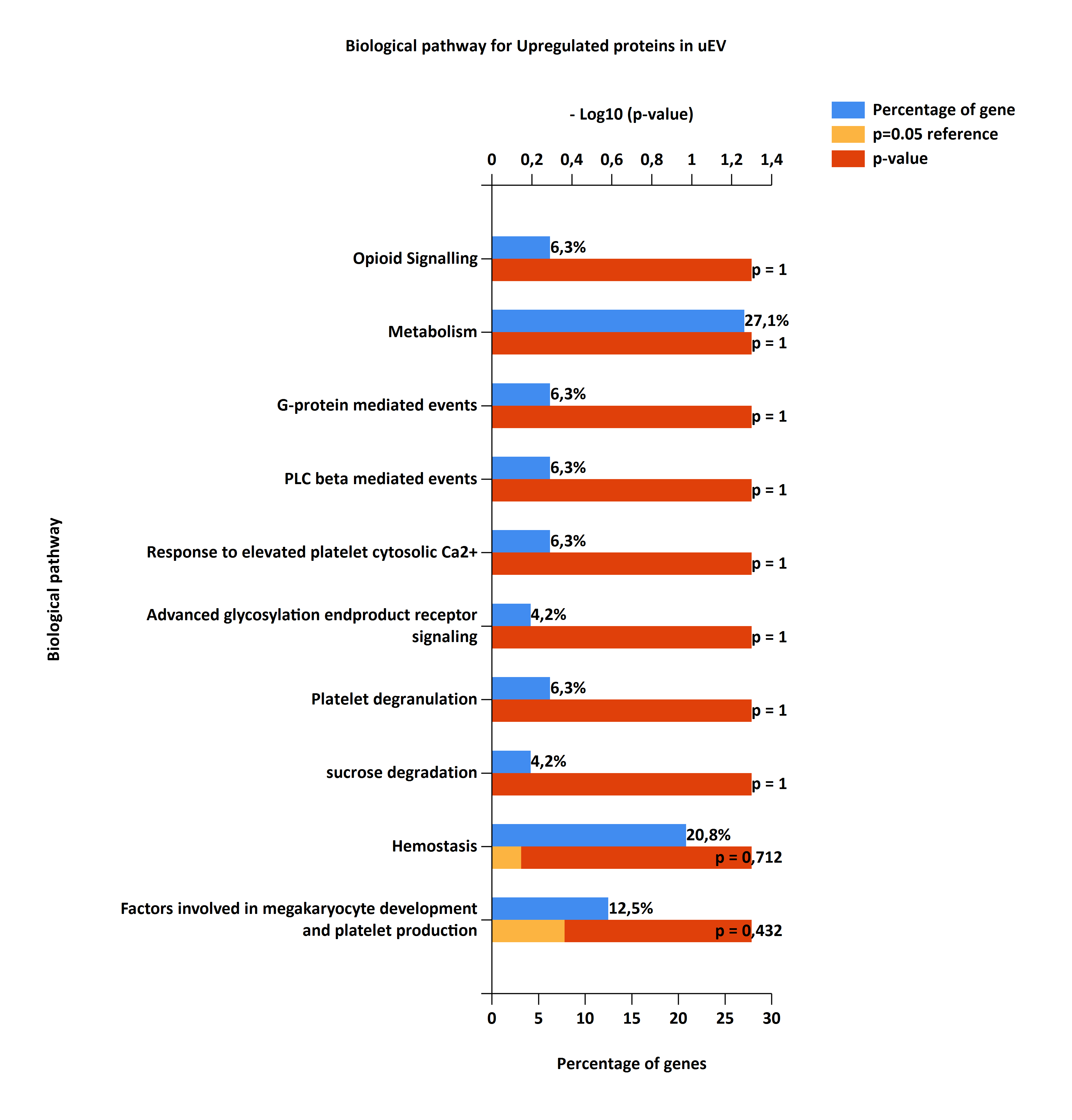

Supplement: Supplementary file 1 [file ijms-23-04344-s001.zip › Fig S2.tiff]
